# Inhibition of mTORC2 component RICTOR impairs tumor growth in pancreatic cancer models

**DOI:** 10.18632/oncotarget.15524

**Published:** 2017-02-20

**Authors:** Katharina M. Schmidt, Claus Hellerbrand, Petra Ruemmele, Christoph W. Michalski, Bo Kong, Alexander Kroemer, Christina Hackl, Hans J. Schlitt, Edward K. Geissler, Sven A. Lang

**Affiliations:** ^1^ Department of Surgery, University Hospital Regensburg, Regensburg, Germany; ^2^ Department of Internal Medicine I, University Hospital Regensburg, Germany; ^3^ Department of Pathology, Hospital of Erlangen, Erlangen, Germany; ^4^ Department of Surgery, University of Heidelberg, Germany; ^5^ Department of Surgery, Technische Universität München (TUM), Munich, Germany; ^6^ Department of Surgery, Universität Freiburg, Freiburg, Germany

**Keywords:** mTORC2/RICTOR, PDAC, AKT, HIF-1

## Abstract

Mammalian Target of Rapamycin complex 2 (mTORC2) and its regulatory component Rapamycin-insensitive companion of mTOR (RICTOR) are increasingly recognized as important players in human cancer development and progression. However, the role of RICTOR in human pancreatic ductal adenocarcinoma (PDAC) is unclear so far. Here, we sought to analyze the effects of RICTOR inhibition in human pancreatic cancer cell lines *in vitro* and *in vivo*. Furthermore, RICTOR expression was determined in human PDAC samples. Results demonstrate that depletion of RICTOR with siRNA (transient knock-down) or shRNA (stable knock-down) has an inhibitory effect on tumor growth *in vitro*. Moreover, RICTOR inhibition led to impaired phosphorylation/activity of AGC kinases (AKT, SGK1). Interestingly, hypoxia-induced expression of hypoxia-induced factor-1α (HIF-1α) was diminished and secretion of vascular-endothelial growth factor-A (VEGF-A) was impaired upon targeting RICTOR. Stable RICTOR knock-down led to significant inhibition of tumor growth in subcutaneous and orthotopic tumor models which was accompanied by significant reduction of tumor cell proliferation. Finally, immunohistochemical analyses of 85 human PDAC samples revealed significantly poorer survival in patients with higher RICTOR expression. In conclusion, these findings provide first evidence for mTORC2/RICTOR as an attractive novel target for treatment of human PDAC.

## INTRODUCTION

Pancreatic ductal adenocarcinoma (PDAC) is one of the most fatal diseases and currently represents the fourth leading cause of cancer-related deaths in the Western world [1]. Moreover, it is projected to be the second leading cause of cancer-related death by 2030 [2]. In contrast to the increasing survival rates for most cancers, advances are poor for PDAC with 5-year overall survival around 7% [1]. To date, surgery for localized disease offers the only curative option (possible in about 20% of patients), increasing the 5-year survival rate up to 25%. On the other hand systemic chemotherapy and/or radiation have only a marginal benefit for survival (reviewed in [3]). Therefore, novel therapeutic approaches for patients suffering from PDAC are urgently needed.

The highly conserved serine/threonine kinase mTOR (mammalian target of rapamycin) represents a crucial regulator of cell growth, proliferation and metabolism, (reviewed by [4, 5]). mTOR is active via two functionally distinct kinase complexes, mTORC1 and mTORC2 (reviewed by [4]). The rapamycin-sensitive mTORC1 with its essential component RAPTOR (regulatory-associated protein of mTOR) mainly regulates protein biosynthesis by phosphorylation of S6K1 and 4E-BP (reviewed by [4, 6]). In contrast, the less well known mTORC2 is characterized by the subunit RICTOR (rapamycin-insensitive companion of mTOR) and acts mainly as a regulator of AGC kinase phosphorylation/activation, including AKT^Ser473^ [4, 6–8]. Functionally, mTORC2 is involved in mediating growth factor signalling, thereby affecting cell survival and cytoskeleton remodelling [4, 7, 9]. Overexpression of RICTOR has been reported in several tumor entities including colorectal cancer, gastric cancer and hepatocellular carcinoma (HCC) [10–12]. Involvement in colorectal cancer metastases [13] and association with poor prognosis in gastric cancer and HCC has also been reported [10, 14]. Furthermore, a recent study in melanoma indicates that RICTOR is frequently overexpressed and cooperates with *NRAS* mutation to stimulate melanoma proliferation [15]. Finally, involvement of mTORC2/RICTOR in cancer metabolism and therapy resistance has been shown in glioblastoma [16, 17]. Hence, mTORC2 and particularly its scaffold protein RICTOR is an interesting target for anti-neoplastic therapy.

Mutations in the PI3K/AKT/mTOR pathway are not a frequently reported alteration in PDAC, whereas the *Ras* oncogene is mutated in over 90% of PDAC patients [3, 18]. Interestingly, a recent report correlates enhanced basal activity of PI3K/AKT/mTOR signalling to resistance of ERK-directed therapy in *Ras*-mutant pancreatic cancer [19]. Although expression of phosphorylated mTOR^Ser2448^ is observed in the majority of PDAC [20], results from clinical trials with mTORC1 inhibitors such as everolimus have been disappointing [21–23]. Indeed, we and others have shown that treatment of pancreatic cancer cells with the mTORC1 inhibitor rapamycin induces AKT^Ser473^ phosphorylation which can be abrogated by simultaneous RICTOR inhibition [24]. In addition, phosphorylation of the mTORC2 target AKT^Ser473^ has been associated with aggressive disease and, more recently, with poor prognosis in PDACs [20, 25]. Therefore, the obvious question is whether mTORC2, and in particular its scaffold protein RICTOR, might serve as an effective target for anti-neoplastic therapy also in PDAC.

In the present study we assessed the role of mTORC2/RICTOR in pancreatic cancer cell lines and human tissue samples. Results demonstrate an impairment of AGC kinases and, surprisingly, an inhibition of hypoxia-inducible factor-1α (HIF-1α) including its downstream target vascular-endothelial growth factor-A (VEGF-A). *In vivo*, RICTOR inhibition led to marked inhibition of tumor growth at least in part via impairment of AKT phosphorylation. Finally, we show that RICTOR is expressed in human PDAC and higher expression is associated with poor prognosis after surgical resection. Hence, the mTORC2/RICTOR is an interesting novel target for anti-cancer therapy in patients suffering from PDAC.

## RESULTS

### Targeting RICTOR in pancreatic cancer cell lines

**Figure 1:**
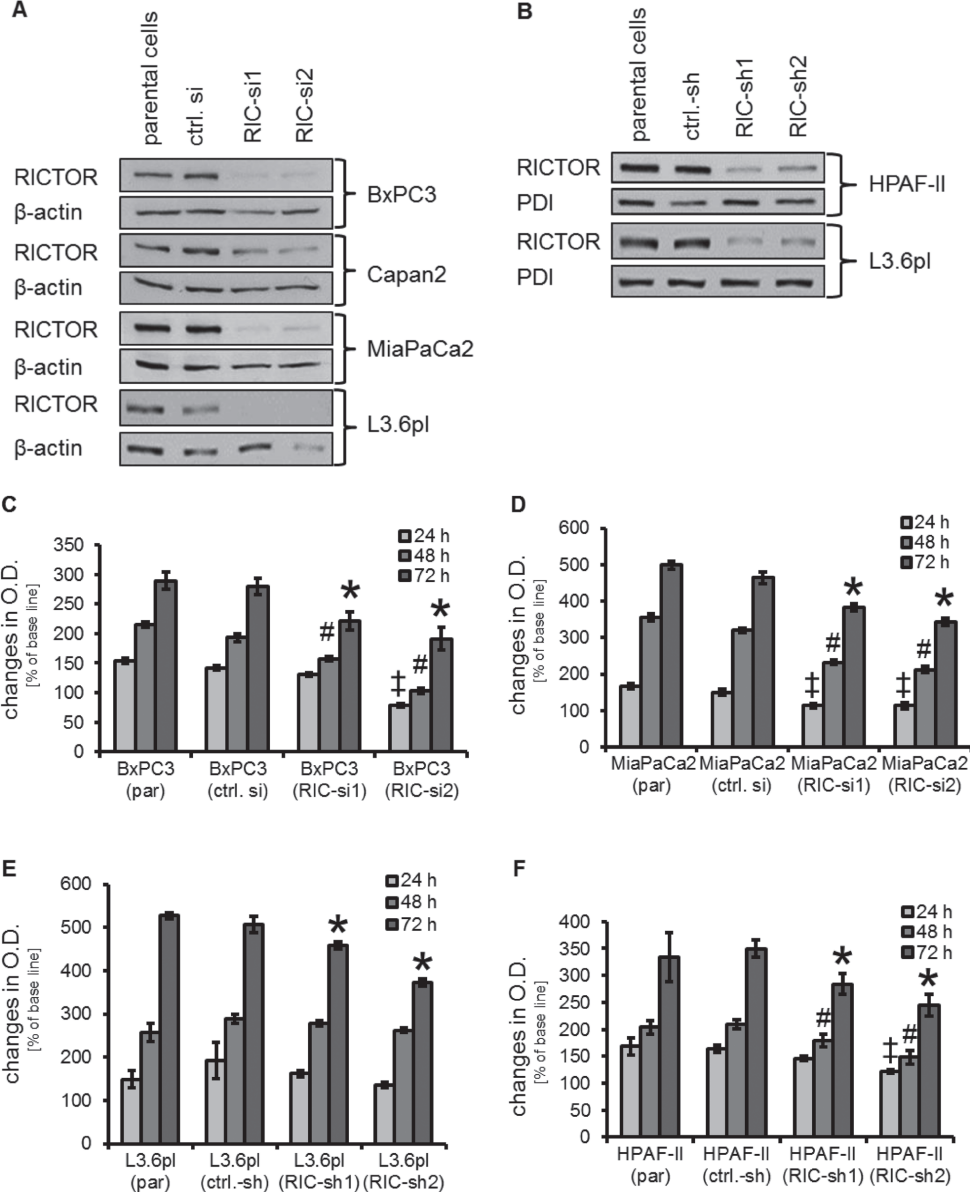
Determination of RICTOR knock-down and its impact on cell growth *in vitro*. (**A**) RICTOR expression is substantially diminished upon transient transfection with siRNA (RIC-si1, RIC-si2) compared to untreated (parental) cells and scrambled siRNA (ctlr. si). (**B**) Stable transfection with two different shRNA-producing plasmids (RIC-sh1, RIC-sh2) impairs RICTOR expression in L3.6pl and HPAF-II cells compared to controls. (**C**) Significant growth inhibition upon transient RICTOR knock-down in BxPC-3 after 48 and 72 h. After 24 h, only RIC-si2 led to growth inhibition (^‡, #, Ù^*p* < 0.05 vs. ctrl. si and par; bars = SEM). (**D**) In MiaPaCa2, significant growth inhibition was found after 24, 48 and 72 h (^‡, #, Ù^*p* < 0.05 vs. ctrl. si and par; bars = SEM). (**E**) Stable knock-down of RICTOR reduces growth of L3.6pl only after 72 h (^Ù^*p* < 0.05 vs. ctrl. si and par; bars = SEM). (**F**) In HPAF-II, growth of cells was impaired after 24 (RIC-si2), 48 and 72 h (^‡, #, Ù^*p* < 0.05 vs. ctrl. si and par; bars = SEM).

Due to the lack of specific inhibitors, RICTOR inhibition was assessed by using either transient or stable siRNA (shRNA, respectively) mediated knock-down. First, RICTOR expression was shown to be strongly inhibited in PDAC cell lines (BxPC3, Capan2, MiaPaCa2 and L3.6pl) after transient transfection with two different siRNAs (Figure 1A). In addition, RICTOR expression upon stable transfection of L3.6pl and HPAF-II cells was strongly diminished (Figure 1B) indicating sufficient inhibition of the target. These observations were confirmed by densitometry from Western blotting showing as knock-down of RICTOR between 47% and 89% (Supplementary Figure 1A–1F).

### Growth inhibition upon RICTOR knock-down *in vitro*

Next, we assessed the effects of RICTOR knock-down on growth of pancreatic cancer cell lines *in vitro*. Upon transient transfection with siRNA, results from MTT assays revealed a minor but significant inhibition of growth after 48 and 72 hours in BxPC3, MiaPaCa2 and L3.6pl cells (Figure 1C, 1D and Supplementary Figure 2A). After 24 hours growth inhibition was only detected in BxPC3 and in MiaPaCa2 (RIC-si2) cells (Figure 1C and 1D). Upon stable RICTOR knock-down, growth impairment was found after 72 hours in L3.6pl and HPAF-II, but only in HPAF-II cells after 48 hours (Figure 1E and 1F). In conclusion, RICTOR knock-down leads to modest, but still significant reduction of growth in pancreatic cancer cell lines.

### Impairment of AGC kinase phosphorylation (activation) by targeting RICTOR

**Figure 2:**
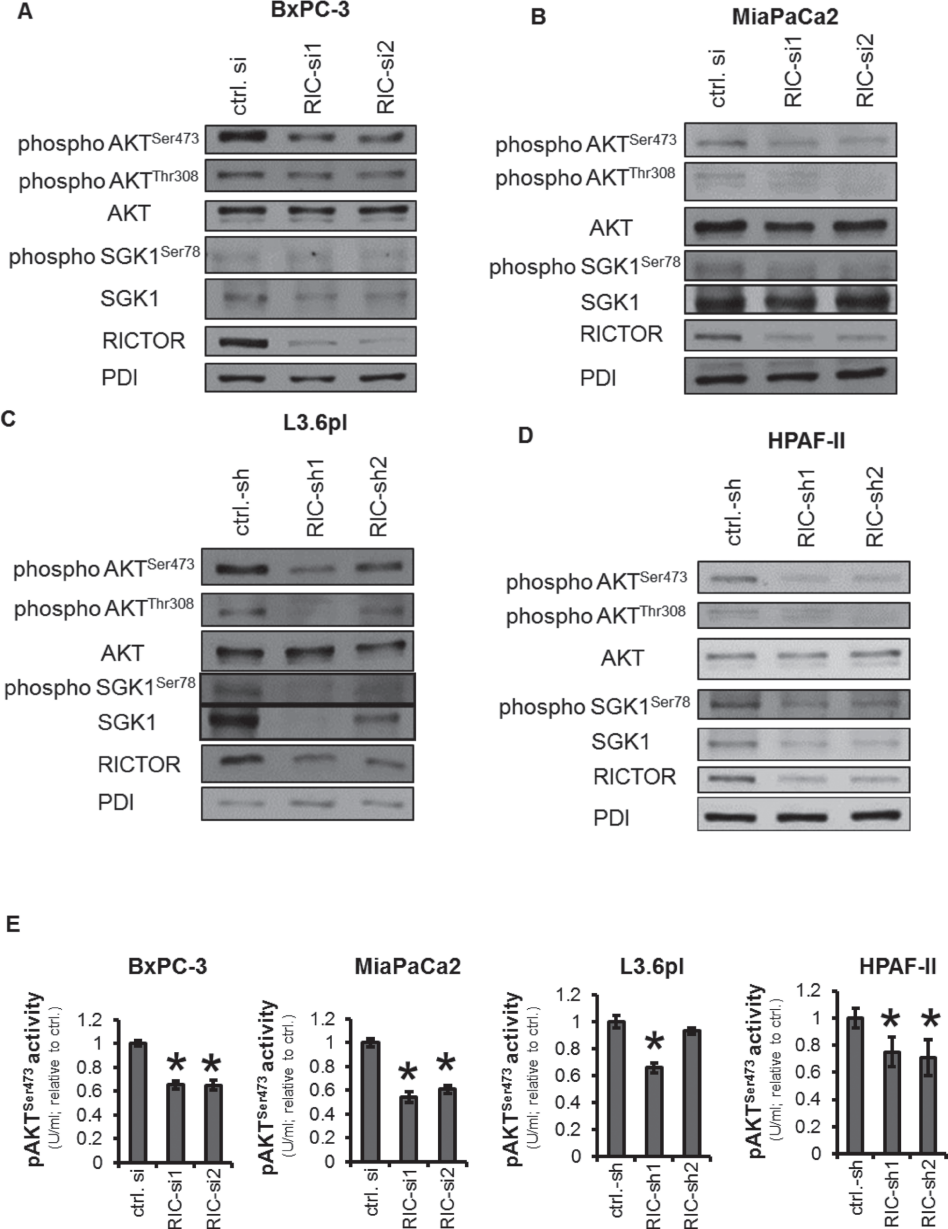
Impairment of AGC kinase upon RICTOR blockade (**A–D**) Phosphorylation of AKT^Ser473^ was diminished in BxPc-3 and MiaPaCa2 (transient RICTOR knock-down) cells as well as in L3.6pl and HPAF-II (stable knock-down) cells. Little or no effect was found on AKT^Thr308^. Moreover, SGK1 expression and phosphorylation at serine 78 was impaired by RICTOR blockade. (**E**) Impairment of RICTOR-mediated reduction of AKT^Ser473^ activity was confirmed by ELISA, except for L3.6pl RIC-sh2. (^Ù^*p* < 0.05 vs. ctrl. si and ctrl.-sh; bars = SEM).

Since AGC kinases are the main downstream target of mTORC2, we assessed the impact of RICTOR blockade on phosphorylation of AKT and SGK1. Consistently, RICTOR blockade reduced AKT^Ser473^ phosphorylation in all cell lines, either upon transient or stable knock-down except for the stable transfected L3.6pl RIC-sh2 clone (Figure 2A–2D, Supplementary Figure 2B). The impairment of AKT^Ser473^ was subsequently confirmed with an activity assay (Figure 2E, Supplementary Figure 2C). In contrast, only minor effects on AKT^Thr308^ phosphorylation were observed after RICTOR blockade (Figure 2A–2D, Supplementary Figure 2B). More interestingly, RICTOR inhibition led to an impairment of SGK1^Ser78^ phosphorylation in MiaPaCa2, L3.6pl and HPAF-II cells, which was accompanied by a reduction of total SGK1 expression except for MiaPaCa2 cells (Figure 2A–2D, Supplementary Figure 2B). Taken together, RICTOR blockade impairs constitutive phosphorylation of AGC kinases in pancreatic cancer cells.

### Inhibition of hypoxia induced HIF-1α expression via RICTOR inhibition

**Figure 3:**
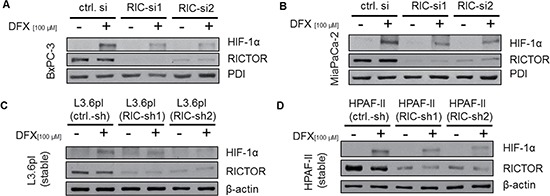
Impact of RICTOR inhibition on HIF-1α expression (**A–D**) DFX (100 μM, 24 h) induces HIF-1α expression in all cell lines. RICTOR blockade with either transient (BxPC-3 (A), MiaPaCa2 (B)) or stable (L3.6pl (C), HPAF-II (D)) knock-down efficiently reduces HIF-1α expression.

Human PDAC is characterized by local hypoxia [26] and hypoxia-inducible factor 1α (HIF-1α) is considered as main transcriptional regulator in reduced oxygen conditions. Therefore, the impact of RICTOR inhibition on HIF-1α expression was determined upon DFX-induced hypoxia. Results show that DFX-induced HIF-1α expression was impaired by transient siRNA mediated RICTOR blockade in BxPC3, MiaPaCa2 and L3.6pl (Figure 3A and 3B, data for L3.6pl not shown). Similarly, stable shRNA mediated RICTOR inhibition diminished HIF-1α expression after DFX induction in L3.6pl and HPAF-II cells (Figure 3C and 3D). In addition, these results were confirmed upon incubation with hypoxia using 1%O_2_ (Supplementary Figure 3A–3D). Hence, targeting the mTORC2 component RICTOR decreases hypoxia-driven HIF-1α expression in pancreatic cancer cell lines and potentially affects factors that are influenced by this transcription factor.

### Impact of RICTOR knock-down on VEGF-A and IL-8 secretion

**Figure 4:**
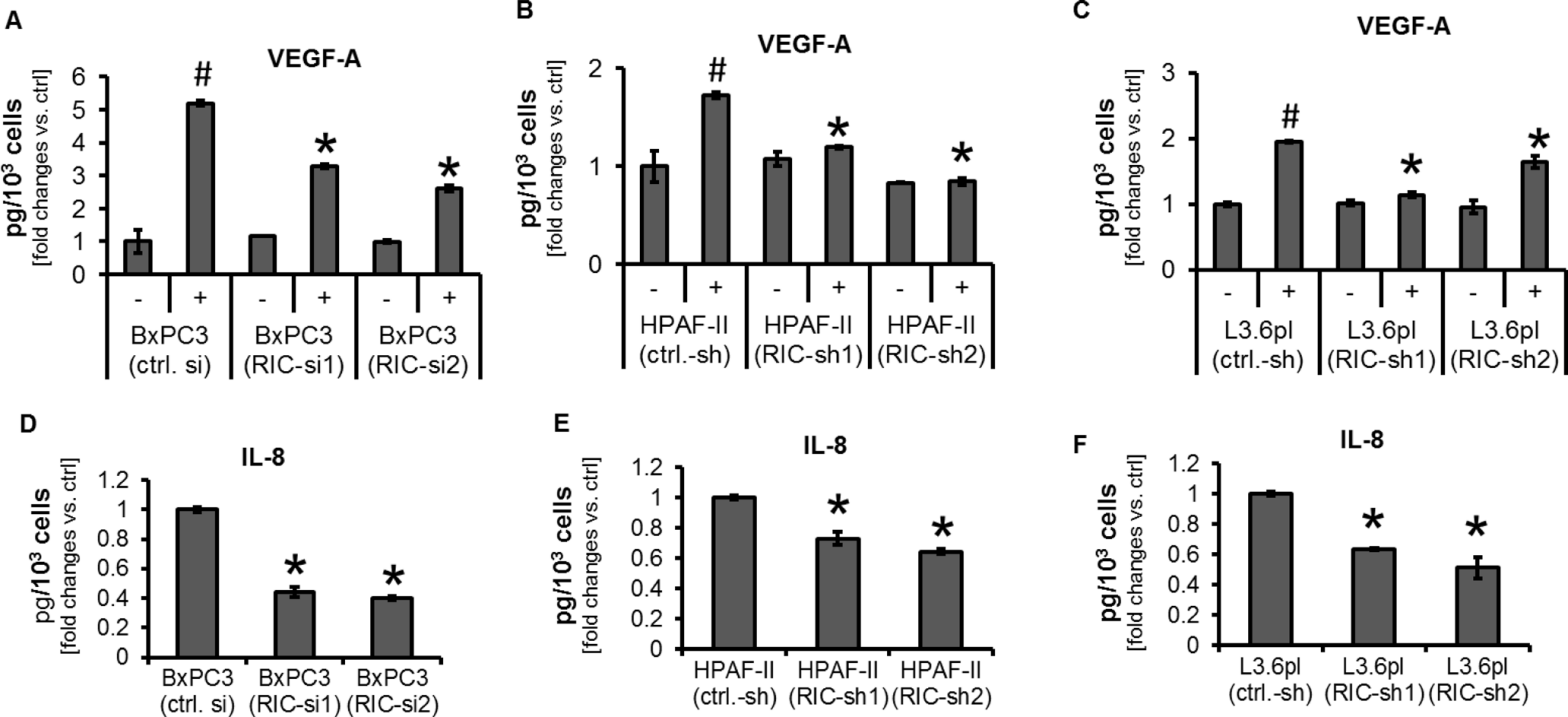
Modulation of VEGF-A and IL-8 secretion upon RICTOR blockade (**A–C**) VEGF-A secretion significantly increases upon induction with DFX (100 μM, 24 h; ^#^*p* < 0.05 vs. untreated ctrl. si; bars = SEM). RICTOR inhibition impairs DFX-induced VEGF-A secretion (^Ù^*p* < 0.05 vs. DFX-treated ctrl. si; bars = SEM). In contrast, RICTOR knock-down did not affect constitutive VEGF-A secretion from BxPC3, HPAF-II and L3.6pl. (**D–F**) Targeting RICTOR led to significant impairment of constitutive IL-8 secretion from BxPC-3, HPAF-II and L3.6pl cells ( ^Ù^*p* < 0.05 vs. respective ctrl. si; bars = SEM).

To confirm the impact of RICTOR blockade on factors affecting the tumor stroma, secretion of VEGF-A and IL-8 was evaluated by ELISA. Pursuant to the modulation of hypoxia-induced HIF-1α expression, VEGF-A secretion from pancreatic cancer cell lines was significantly reduced upon RICTOR inhibition after incubation with DFX (Figure 4A–4C, Supplementary Figure 2D and Supplementary Figure 4A). These effects were also observed when cells were cultured upon hypoxic conditions using 1% O_2_ (Supplementary Figure 5A–5D). No effect on constitutive VEGF-A secretion was observed. In contrast, RICTOR blockade led to significant reduction of constitutive IL-8 secretion from cancer cell lines (Figure 4D–4F), whereas no induction of IL-8 upon incubation with DFX was found (data not shown). In summary, RICTOR inhibition impairs secretion of hypoxia-induced VEGF-A secretion and constitutive IL-8 secretion from pancreatic cancer cell lines which potentially affects the surrounding tumor stroma.

### Targeting RICTOR reduces tumor growth in subcutaneous models

**Figure 5:**
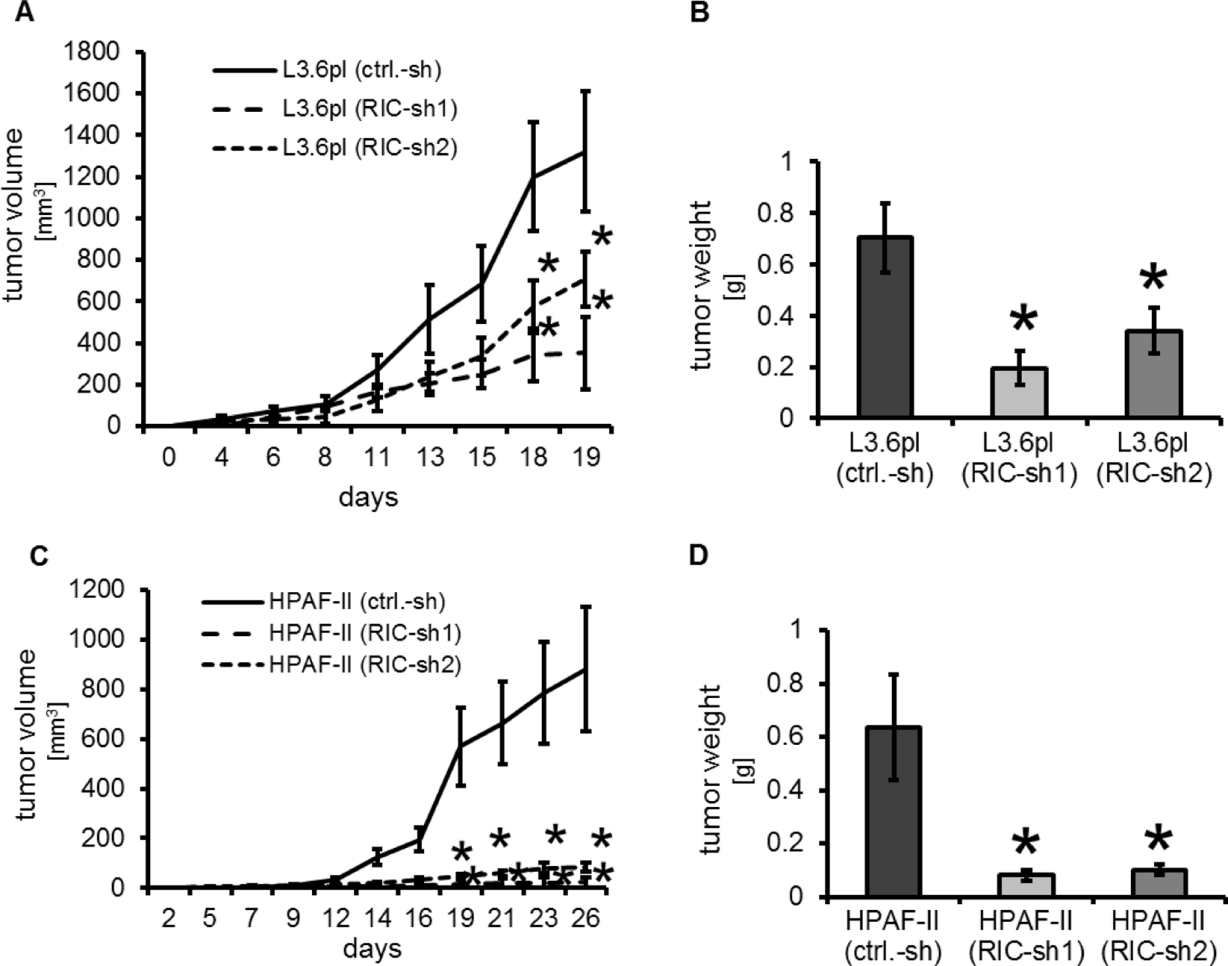
Targeting RICTOR in subcutaneous tumor models (**A**) RICTOR knock-down significantly impairs subcutaneous tumor growth of L3.6pl pancreatic cancer cells *in vivo* (^Ù^*p* < 0.05 vs. ctrl.-sh; bars = SEM). (**B**) Final tumor weight confirmed significant impairment of tumor growth (^Ù^*p* < 0.05 vs. ctrl.-sh; bars = SEM). (**C**) Similar, results were obtained with HPAF-II pancreatic cancer cells (^Ù^*p* < 0.05 vs. ctrl.-sh; bars = SEM). (**D**) This significant difference was also evident regarding final tumor weight (^Ù^*p* < 0.05 vs. ctrl.-sh; bars = SEM).

Next, the *in vitro* results were validated in a subcutaneous mouse model using stable transfected L3.6pl cells [L3.6pl (ctrl-sh), L3.6pl (RIC-sh1), L3.6pl (RIC-sh2)]. Stable knock-down of RICTOR led to significant inhibition of tumor volume (Figure 5A) which was also reflected by decreased final tumor weights (Figure 5B). Results were subsequently confirmed with stable transfected HPAF-II cells [HPAF-II (ctrl-sh), HPAF-II (RIC-sh1), HPAF-II (RIC-sh2)] to rule out cell line-specific effects. Similar to the effects in L3.6pl cells, RICTOR knock-down led to markedly decreased tumor volumes (Figure 5C) and weights (Figure 5D). From these results we conclude that targeting RICTOR has growth inhibitory effects on pancreatic cancer cells *in vivo*.

### Inhibition of RICTOR impairs orthotopic tumor growth via effects on proliferation and vascularization

**Figure 6:**
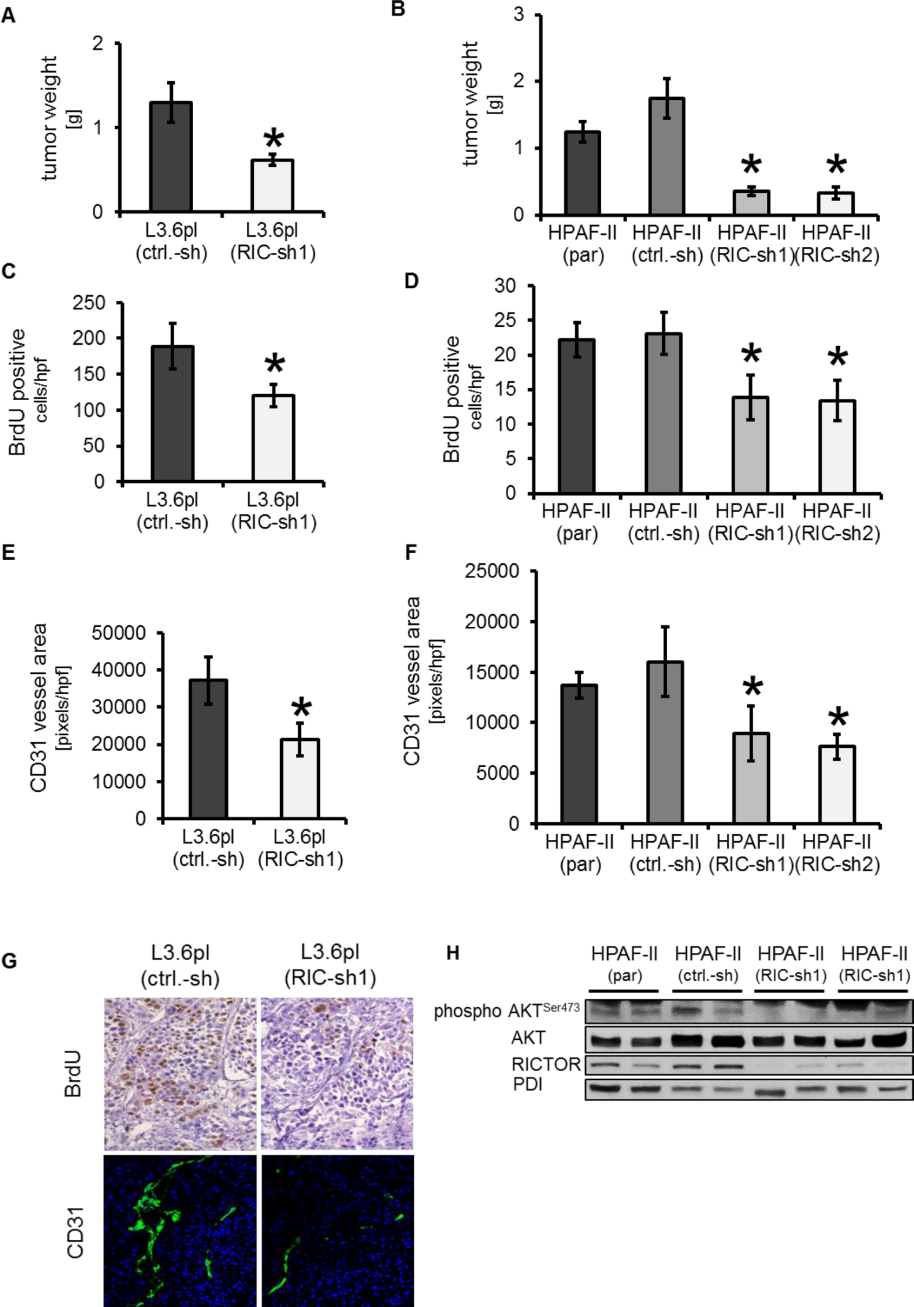
RICTOR knock-down in orthotopic tumor models (**A, B**) Targeting RICTOR leads to significant impairment of tumor growth in orthotopic pancreatic cancer models using L3.6pl (A) and HPAF-II cells (B) (^Ù^*p* < 0.05 vs. ctrl.-sh in L3.6pl and *p < 0.05 vs. ctrl.-sh and par in HPAF-II cells; bars = SEM). (**C, D**) RICTOR knock-down significantly impairs tumor cell proliferation as determined by BrdU staining in both orthotopic models (*p < 0.05 vs. ctrl.-sh in L3.6pl and *p < 0.05 vs. ctrl.-sh and par in HPAF-II cells; bars = SEM). (**E, F**) Assessment of tumor vascularization reveals a significant reduction of CD31 vessel area upon RICTOR inhibition (*p < 0.05 vs. ctrl.-sh in L3.6pl and *p < 0.05 vs. ctrl.-sh and par in HPAF-II cells; bars = SEM). (**G**) Examples are shown for BrdU and CD31 from RICTOR knock-down (right side) and controls (left side). (**H**) RICTOR knock-down *in vivo* was confirmed by Western blotting. Similar, inhibition of AKT^Ser473^ phosphorylation was determined. Results are shown for HPAF-II cells; similar results were obtained from L3.6pl cells.

To assess our findings in an appropriate microenvironment, we subsequently used orthotopic tumor models. First L3.6pl [L3.6pl (ctrl-sh), L3.6pl (RIC-sh1)] were used to address this issue, showing that RICTOR inhibition significantly impairs the final tumor volume as determined upon necropsy on day 25 after tumor cell injection (Figure 6A). Subsequently, these results were confirmed with HPAF-II cells [HPAF-II (ctrl-sh), HPAF-II (RIC-sh1), HPAF-II (RIC-sh2)]. In this experiment a control without any manipulation (parental HPAF-II cells) was included to ensure that the empty vector control had no effects on tumor growth [HPAF-II (par)]. Similar to the previous results from L3.6pl, a significant growth inhibition as reflected by final tumor weight (day 28) was found upon RICTOR inhibition (Figure 6B). Assessment of visible metastases showed a significant reduction of enlarged lymph nodes in the L3.6pl model which was also observed in HPAF-II. Regarding liver metastases, a similar trend towards fewer liver metastases was found in the L3.6pl model; HPAF-II do not form liver metastases in our hands (Table 1). Next we analyzed the impact on tumor cell proliferation by BrdU staining. Results demonstrated a significant reduction of BrdU positive tumor cells in the RICTOR knock-down groups in both experiments (Figure 6C, 6D, 6G). CD31 vessel area was determined to assess effects on tumor vascularization. The rational is based on our observation that RICTOR blockade impairs secretion of VEGF-A and HIF-1α. In line with our *in vitro* results (Figure 3 and 4), RICTOR blockade reduced tumor vascularization versus respective controls (Figure 6E–6G). RICTOR knock-down and inhibition of AKT^Ser473^ phosphorylation *in vivo* was confirmed by Western blotting (Figure 6H). In conclusion, results from our experiments demonstrate that RICTOR inhibition impairs tumor growth from pancreatic cancer cell lines at least in part via effects on proliferation and vascularization.

**Table 1:** Number of liver and lymph node metastases in the orthotopic tumor models

L3.6pl orthotopic model				
	Liver metastases		LN metastases	
	positive	negative	positive	negative
L3.6pl (ctrl.-sh) (*n* = 10)	6 (60%)	4 (40%)	8 (80%)	2 (20%)
L3.6pl (RIC-sh)1(*n* = 10)	2 (20%)	8 (80%)	1 (10%)	9 (90%)*

HPAF-II orthotopic model^1^		
	LN metastases	
	positive	negative
HPAF-II (par) (*n* = 8)	6 (75%)	2 (25%)
HPAF-II (ctrl.-sh) (*n* = 8)	5 (62.5%)	3 (37.5%)
HPAF-II (RIC-sh1) (*n* = 5)	1 (20%)	4 (80%)
HPAF-II (RIC-sh2) (*n* = 5)	1 (20%)	4 (80%)

^1^no liver metastases observed.

*p < 0.05 vs. control.

### RICTOR expression in human pancreatic ductal adenocarcinoma (PDAC)

**Figure 7:**
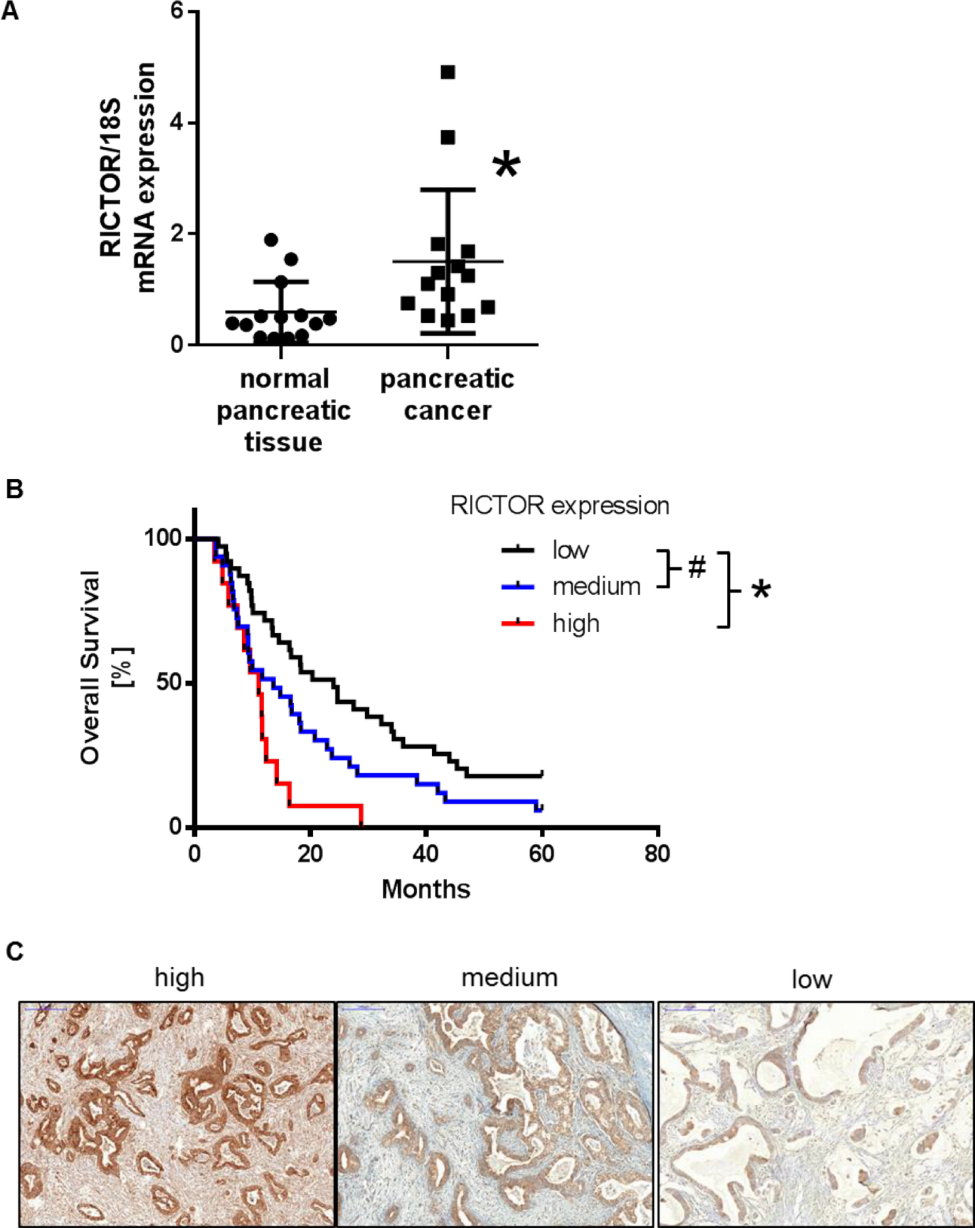
Expression of RICTOR in human pancreatic ductal adenocarcinoma (**A**) Expression of RICTOR mRNA is significantly higher in PDAC compared to normal pancreas (^Ù^*p* = 0.02) (*n* = 13/group). (**B**) Patients with low RICTOR expression (black line; *n* = 39) have significantly better median survival compared to medium (blue; *n* = 33; ^#^*p* = 0.04) or high (red; *n* = 13; *p < 0.0001) expression. (**C**) Examples for high, medium and low RICTOR expression in human PDAC.

Finally, we assessed the relevance of the mTORC2 component RICTOR in human PDAC samples. Higher levels of RICTOR mRNA were detected in samples from PDAC compared to normal pancreatic tissue (Figure 7A). Moreover, we determined the expression of RICTOR in 85 samples from patients that underwent surgery for pancreatic cancer by immunohistochemistry. This analysis revealed a significantly shorter patient survival with high (median survival of 11.1 months) or medium (median survival 13.6 months) expression compared to low RICTOR expression (median survival 24 months) (Figure 7B and 7C). These results indicated that expression of the mTORC2 component RICTOR is associated with reduced survival in patients with PDAC and, therefore, might serve as an interesting target for anti-cancer therapy.

## DISCUSSION

PDAC remains devastating with overall 5-year survival far below 10%, mandating novel therapeutic approaches to improve the prognosis for these patients. In the present study we found that inhibition of the mTORC2 component RICTOR impairs activation of signaling pathways (AKT, SGK1), reduces expression of hypoxia-induced HIF-1α and diminishes secretion of critical cancer-promoting factors involved in stromal recruitment such as VEGF-A and IL-8. Moreover, *in vivo* tumor growth was significantly decreased in subcutaneous and orthotopic models upon RICTOR knock-down. Finally, we showed that higher RICTOR expression is associated with a poor prognosis in patients after resection of PDAC. These results provide the first evidence that RICTOR is an attractive new target for PDAC therapy.

Clinical studies to date have shown little or no benefit in PDAC by using traditional mTOR inhibitors [21, 22]. Notably, however, research thus far has primarily focused on inhibition of the rapamycin sensitive mTORC1 using so called rapalogs. Here, we have taken another approach to PDAC by blocking RICTOR, which is a key component of the rapamycin insensitive mTORC2. RICTOR inhibition showed impairment of AGC kinase activation including AKT^Ser473^ in pancreatic cancer cell lines. Indeed, expression of AKT^Ser473^ in PDAC has been associated with poor prognosis [20] and AKT is known to be involved in regulation of several events that contribute to tumor growth such as survival, apoptosis, tumor metabolism and chemosensitivity [27]. Interestingly, results from our study show only moderate impairment of tumor growth *in vitro*, which is in contrast to previous reports where mTORC2/RICTOR inhibition was associated with impairment of *in vitro* growth in several cancer entities [28–31]. With regard to PDAC, other signaling pathways such as Kras/Mek/Erk or src may also play a critical role in the proliferation and growth of pancreatic cancer cells, providing a partial explanation for the modest effects of RICTOR inhibition [32, 33]. In this respect we found that expression of SGK1 (also an AGC kinase) is impaired upon RICTOR blockade, which is likely related to the impairment in IL-8 secretion from tumor cells [34]. Therefore, results from our study clearly demonstrate that AGC kinases can be inhibited in PDAC cells upon targeting RICTOR.

In our study we found that RICTOR blockade results in inhibition of hypoxia-induced HIF-1α expression. This observation correlates with a significant reduction in VEGF-A which is involved in the recruitment of stromal cells. Similar to AKT, HIF-1α expression has been associated with poor prognosis and early recurrence in PDAC [35]. Hypoxic zones with subsequent stabilization of HIF-1 are very common in PDAC [26, 33, 35]. In the context of hypoxia, we and others have described HIF-1α inhibition upon targeting mTORC1 with rapamycin [36–38]. However, to our knowledge, impairment of hypoxia-induced HIF-1α expression and VEGF-A secretion upon RICTOR blockade in cancer cell lines has not been reported so far. Nonetheless, inhibition of constitutive HIF-1α expression in renal cell carcinoma cell lines, mouse embryonic fibroblasts (MEF), 293 cells [39, 40], and significant reduction of constitutive VEGF-A production in a retinal pigmental epithelial cell line has been described [41]. Indeed, RICTOR has been shown to impair c-Myc expression in glioblastoma cells [16] and c-Myc is known to interact with HIF-1α in cancer cells (reviewed by [42]). Moreover, c-Myc has been described to down-regulate IL-8 in chronic liver disease [43]. Therefore, one might speculate that the connection between RICTOR/c-Myc on the one hand and c-Myc/HIF-1 on the other hand is responsible for the observed effects on hypoxia-induced HIF-1α and VEGF-A upon RICTOR blockade. Moreover, the interplay might also explain the reduction of constitutive IL-8 secretion. Further research is warranted to clarify this. Nonetheless, our findings show that inhibition of RICTOR impairs hypoxia-induced HIF-1α in addition to the impairment of AGC kinases, further strengthening the rational for targeting RICTOR in PDAC.

Assessment of RICTOR blockade *in vivo* demonstrated a significant inhibition of subcutaneous tumor growth and development of orthotopic PDAC xenografts. Impairment of tumor growth by RICTOR blockade *in vivo* has been described for other cancer entities such as colon cancer and malignant pheochromocytoma [30, 44]. Nonetheless, our study is the first that addresses this issue in PDAC. Interestingly, growth inhibition in our model was associated with strong impairment of tumor cell proliferation as determined by BrdU staining, even though only moderate reduction of tumor growth was detected *in vitro*. This is in contrast to results from Carr and coworkers who showed that depletion of RICTOR in established skin tumors led to tumor regression without changes in tumor cell proliferation [45]. However, mTORC2 has recently been implicated in the regulation of tumor metabolism [46], showing involvement in aerobic glycolysis (Warburg effect) for glioblastoma [16]. Effects on tumor metabolism could be linked to inhibition of AKT and HIF-1α phosphorylation/expression by RICTOR blockade that we observed; since both factors are key players in metabolic reprogramming (reviewed by [47]). Further research is currently ongoing to assess this issue.

Importantly, we determined that high RICTOR expression in resected PDAC is associated with poor survival. Similar results have been reported for other tumor entities such as gastric cancer and HCC but so far not for PDAC [10, 12]. Over the recent years several subgroups of PDAC that might benefit from mTOR inhibition have been reported, but these reports focused primarily on mTORC1 [33, 48]. In contrast, Kennedy *et al*. reported that expression of the mTORC2/RICTOR down-stream target pAKT^Ser473^ is associated with poor prognosis in PDAC [20]. Moreover, a recently published study defines molecular subtypes of pancreatic cancer showing that the squamous subtype is, among others, characterized by gene networks involved in response to hypoxia, metabolic reprogramming and Myc pathway activation [49]. Since HIF-1 and AGC kinases are involved in these processes, RICTOR inhibition might be a promising option for anti-neoplastic therapy in PDAC. In addition, the squamous tumor subtype was enriched for activated integrin and EGF signaling, both known to be influenced by mTORC2/RICTOR [29, 49, 50]. Finally, a recent study reports that glucose-dependent acetylation of RICTOR confers resistance to targeted therapy in glioma [17]. Since hyperglycemia is commonly found in PDAC, this mechanism might play an additional role in RICTOR's protumorigenic effects in PDAC. In summary, the data we show in our study provides strong evidence that targeting RICTOR is an interesting option for anti-neoplastic therapy in PDAC.

## MATERIALS AND METHODS

### Cell lines, culture conditions and reagents

The human pancreatic cancer cell lines BxPC3, Capan2, HPAF-II, MiaPaCa2 (ATCC, Manassas, VA, USA) and L3.6pl (kindly provided by I.J. Fidler, The University of Texas, M.D. Anderson Cancer Center) were used for the experiments. BxPC3 cells were cultured in Roswell Park Memorial Institute Medium (RPMI; Lonza Group, Basel, Switzerland) supplemented with 10% fetal calf serum (FCS; Sigma-Aldrich, St. Louis, MO, USA); Capan2, HPAF-II, L3.6pl, and MiaPaca2 were cultured in Dulbecco's Modified Eagle's Medium (DMEM; Lonza Group, Basel, Switzerland) supplemented with 10% FCS. All cell lines were maintained at 37°C in a humidified atmosphere with 5% CO_2_.

### Transient and stable suppression of RICTOR

Due to the lack of specific inhibitors, we used RNA interference for RICTOR knock-down. BxPC3, Capan2, MiaPaCa2, and L3.6pl were transiently transfected with two different siRNA sequences targeting RICTOR and scrambled siRNA control (Silencer^®^ Select, s48410 (=RIC-si1), s226000 (=RIC-si2), scrambled si = ctrl. si; Invitrogen, Waltham, MA, USA) using Lipofectamine RNAiMAX transfection reagent (Invitrogen, Waltham, MA, USA). Pre-plated cells were incubated with transfection mixture (Opti-MEM^®^ (Gibco, Waltham, MA, USA), Lipofectamine 2.5 % vol/vol, siRNA 50 nM) for 6 hours. 48 hours after transfection cells were processed for further experiments as well as for confirmation of knock-down efficiency by Western blotting.

Stable transfection in L3.6pl and HPAF-II cells was performed with two different anti-RICTOR shRNA (RIC-sh1; RIC-sh2) and one empty vector control (ctrl.-sh) (Sure Silencing, Quiagen, Hilden, Deutschland) using Lipofectamine 2000 transfection reagent (Invitrogen, Waltham, MA, USA). Cells were cultivated and expanded in selective medium containing neomycin (Sigma Aldrich, Deisenhofen, Germany) for HPAF-II. Impairment of RICTOR expression was confirmed by Western blotting.

### Determination of cell growth by 3-(4,5-dimethylthiazol-2-yl)-2,5-diphenyltetrazolium (MTT) bromide assays

To evaluate the effects of RICTOR inhibition on tumor growth *in vitro*, 2 × 10^3^ cancer cells with transient or stable RICTOR knock-down were seeded into 96-well plates. MTT assays were performed as described [51].

### Western blotting

Western blots were performed to confirm RICTOR knock-down and its impact on AGC kinases and transcription factor HIF-1α. Whole-cell lysates were prepared as described elsewhere [52]. For analyzing HIF-1α expression, cells were treated with the hypoxia mimic desferroxamine (DFX, Sigma-Aldrich, St. Louis, MO, USA; 100 μM) or cultured in 1%O_2_ for 24 hours before lysis. Protein from tumor tissue was prepared as described earlier [52]. Protein samples (30 μg) were subjected to a denaturating 10% SDS-PAGE. Sequentially, the membranes were probed with primary antibodies against RICTOR, pAKT^Ser473^, pAKT^Thr308^, AKT, SGK-1, pSGK1^Ser78^ (Cell Signaling, Beverly, MA, USA) and HIF-1α (Novus Biological, Littleton, CO, USA). β-actin (Santa Cruz Biotechnologies, Dallas, TX, USA) and PDI (Biomol, Hamburg, Germany) served as loading control. Antibodies were detected by enhanced chemoluminescence (Sigma-Aldrich, St. Louis, MO, USA).

### AKT^Ser473^ activity

An enzyme immunometric assay (R&D Bioscience) was used to determine AKT^Ser473^ activity as described [38]. Pancreatic cancer cells (1.5 × 10^3^ cells) with either transient or stable RICTOR knock-down were cultured for 24 hours. Analyses of cell lysates were performed according to the manufacturers’ protocol.

### Enzyme-linked immunosorbent assay (ELISA) for VEGF-A and IL-8

To assess changes in VEGF-A and IL-8 secretion upon RICTOR blockade, commercial ELISA kits were used according to the manufacturer's instruction (Human VEGF Quantikine ELISA Kit, Human CXCL8/IL-8 Quantikine ELISA Kit, R&D Systems, Minneapolis, MN, USA). Cells were plated at 40–50% density and exposed to DFX (100 μM) or cultured in 1%O_2_ for 24 hours before collecting culture supernatants.

### Subcutaneous and orthotopic tumor models

Eight-week old athymic nude mice (CAnN.Cg-Foxn1nu/Crl, Charles River, Sulzfeld, Germany) were used to assess the impact of RICTOR blockade *in vivo*. Experiments were approved by the Institutional Animal Care and Use Committee of the University of Regensburg and the regional authorities. In addition, experiments were conducted according to “Guidelines for the Welfare of Animals in Experimental Neoplasia” published by The United Kingdom Coordinating Committee on Cancer Research.

First, RICTOR blockade was assessed in subcutaneous tumor models. 5 × 10^5^ cells with stable RICTOR knock-down and respective control cells with empty vector (L3.6pl (RIC-sh1), L3.6pl (RIC-sh2), L3.6pl (ctrl-sh); HPAF-II (RIC-sh1), HPAF-II (RIC-sh2), HPAF-II (ctrl-sh)) were injected into the right flank (*n* = 6–7/group) as described before [53]. Tumor diameters were measured and volumes calculated (*width*^2^ *x length x 0.5)*. The experiment was terminated after 19 days (L3.6pl) and 26 days (HPAF-II), respectively. Tumors were excised, weighed and processed for further experiments.

Next, effects of RICTOR blockade were determined in orthotopic tumor models. 1x 10^6^ L3.6pl cells (ctrl-sh, RIC-sh1) with stable RICTOR knock-down were injected into the pancreatic tail of nude mice (*n* = 10/group), and the experiment was terminated after 25 days. Tumors were excised, weighed and processed for further analyses. Finally, results from the orthotopic L3.6pl tumor model were confirmed in HPAF-II cells. For this experiment, a control group injected with native HPAF-II cells was included in addition to empty vector controls [HPAF-II (par), HPAF-II (ctrl-sh)]; moreover, two clones with a different shRNA sequence for RICTOR knock-down were employed [HPAF-II (RIC-sh1), HPAF-II (RIC-sh2); *n* = 5–8 mice/group]. The experiment was terminated after 28 days and tumors were processed similar to the orthotopic L3.6pl model. In both models, macroscopically visible metastases (liver, lymph nodes) were determined upon necropsy.

### Immunohistochemical analysis of tumor cell proliferation and tumor vascularization

To investigate the effects of impaired RICTOR expression on tumor cell proliferation *in vivo*, the thymidine-analogue bromodeoxyuridine (BrdU; Sigma Aldrich; 1 mg/mouse) was injected intraperitoneally 12 hours before termination. BrdU uptake was visualized by staining BrDU on cryosections of tumor tissue by using a commercially available kit (BD Biosciences, San Jose, CA, USA) as described elsewhere [54]. BrdU-positive tumor cells were counted in 4 high-power fields (hpf) per tumor section at 20x magnification and averages were calculated.

Impact on tumor vascularization was determined by CD31 immunostaining. In brief, cryosections of tumor tissue were fixed in cold acetone and chloroform, washed with PBS and exposed to the primary antibody against CD31 (1:50; Pharmingen, Heidelberg, Germany); the secondary antibody AlexaFluor 488 (1:200; Live Technologies, Carlsbad, CA, USA) was applied as described [51]. Images were taken in four different quadrants of each tumor section at 20x magnification. The CD31 positive vessel area was determined by converting images to grayscale and setting a consistent threshold for all slides using ImageJ software (version 1.49, NIH). Vessel area is shown as pixels per hpf.

### Assessment of RICTOR in patient samples

First, RICTOR mRNA expression in normal pancreatic tissue was compared to tissue from PDAC (*n* = 13/group). The study was approved by IRB of the Technische Universität München (TUM), Munich, Germany (5892/13). In brief, 30 mg of tissue was minced and total RNA was isolated and processed by spin column-based nucleic acid purification (NucleoSpin^®^ RNA II, Macherey-Nagel, Düren, Germany). From each sample, a 1 μg aliquot was reversely transcribed into cDNA (Roche, Mannheim, Germany). Primer pairs were as follows: RICTOR (5′-agtgaatctgtgccatcgagt; 3′-agtagagctgctgccaaacc) and 18S (5′- gtaacccgttgaaccccatt; 3′-ccatccaatcggtagtagcg). These primers were optimized for MgCl_2_ and annealing, and PCR products were confirmed by gel electrophoresis. RT-PCR was performed using the LightCycler system and LightCycler^®^ 480 SYBR Green I Master kit (Roche, Mannheim, Germany).

Subsequently, RICTOR expression in human PDAC was determined in tumor specimens from 85 patients who underwent surgery for PDAC with curative intend at the Department of Surgery, University Hospital Regensburg, Germany between 2001 and 2008. The study was approved by the local IRB, University Hospital Regensburg, Germany (15-101-0055). Representative formalin-fixed, paraffin-embedded tissue sections were immunostained using a RICTOR-specific antibody (Cell Signaling, Beverly, MA, USA). Slides were deparaffinized in xylene, followed by treatment with a graded series of alcohol washes [100%, 96%, 70% ethanol/ddH_2_O (vol/vol)], rehydration in phosphate-buffered saline (pH 7.5) and blocked against endogenous peroxidase with H_2_O_2_. Slides were incubated with primary antibody (1:25 dilution) at 4°C overnight. After washing with phosphate-buffered saline, biotinylated antibody (1:50 dilution; Vectastain Universal Elite ABC Kit, Vector Laboratories, Burlingame, CA) was added to tissue sections. RICTOR was visualized by Vectastain Elite ABC reagent (Vector Laboratories), followed by incubation with DAB. Nuclei were counterstained with hematoxylin. Negative controls were performed by omitting the primary antibody. Finally, the cytoplasmatic immunoreactivity for RICTOR was rated on a scale from 1 to 3 according to a modification of an established scoring system [11].

### Statistical analysis, densitometry and survival data

Statistical analyses were carried out by using SigmaStat (Version 3.0). Results of *in vivo* experiments were analyzed for significant outliers using the Grubb's test (www.graphpad.com). Tumor-associated variables of *in vivo* experiments were tested for statistical significance using the Mann-Whitney *U* test for nonparametric data or ANOVA followed by Tukey's multiple comparison tests for more than 2 groups. The two-sided Student's *t* test was applied for analysis of *in vitro* data. Patient survival was determined using the Cox regression analyses. All results are expressed as the mean ± standard error of the mean (SEM). Densitometry was performed using Image J (1.46r).

## SUPPLEMENTARY MATERIALS FIGURES


