# Supplementary material for: Inhibition of mTORC2 component RICTOR impairs tumor growth in pancreatic cancer models
Source: Oncotarget. 2017 Feb 20;8(15):24491–505. doi: 10.18632/oncotarget.15524 (PMC5421865; doi:10.18632/oncotarget.15524)
Supplement: Supplementary file 1 [file oncotarget-08-24491-s001.pdf]

Inhibition of mTORC2 component RICTOR impairs tumor growth in pancreatic cancer models

Supplementary Materials

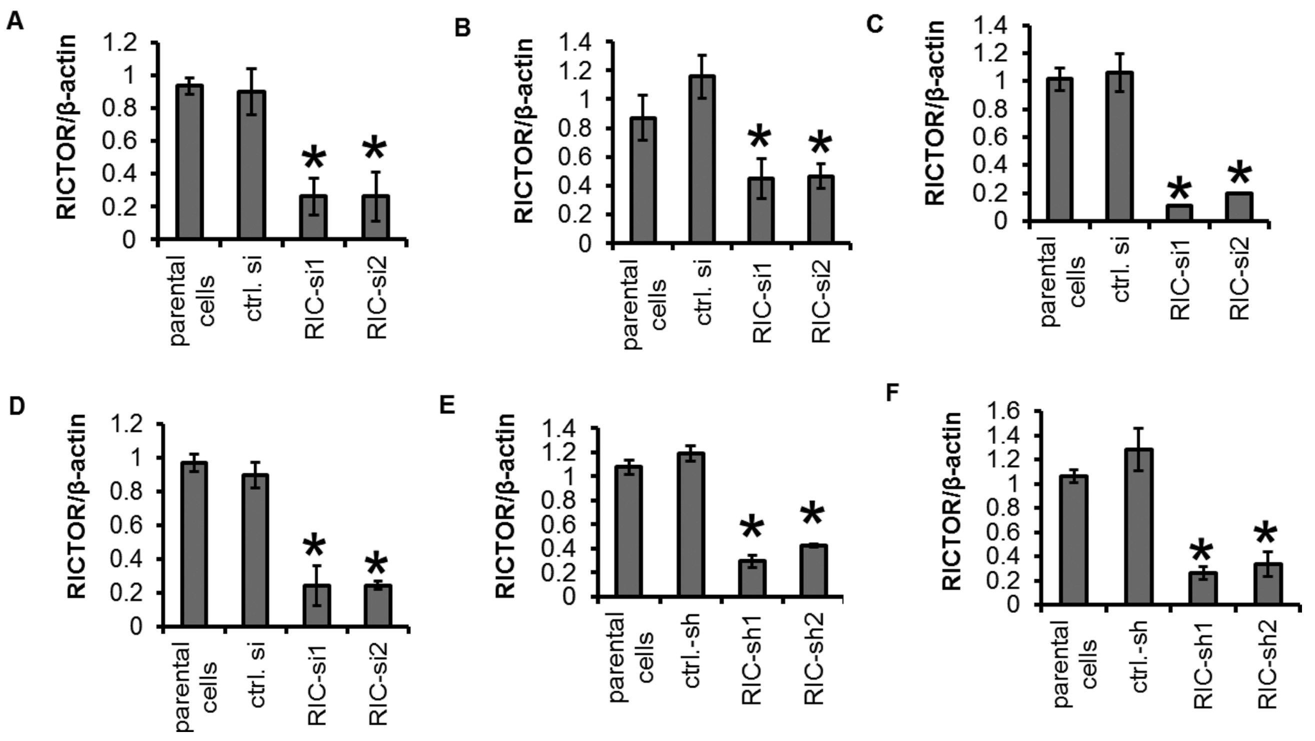

Supplementary Figure 1: Densitometry of RICTOR expression upon transient and stable knock-down. (**A–D**) Transient transfection with RICTOR siRNA significantly impairs RICTOR expression in relation to β-actin (loading control) in human pancreatic cancer cell lines BxPC3, Capan2, MiaPaCa2 and L3.6pl. (**E** and **F**) Stable transfection with RICTOR shRNA also significantly impairs RICTOR expression in relation to loading control in human pancreatic cancer cell lines HPAF-II and L3.6pl. Results are calculated from 3 independent experiments (*$p < 0.05$ vs. ctrl. si and par; bars = SE)

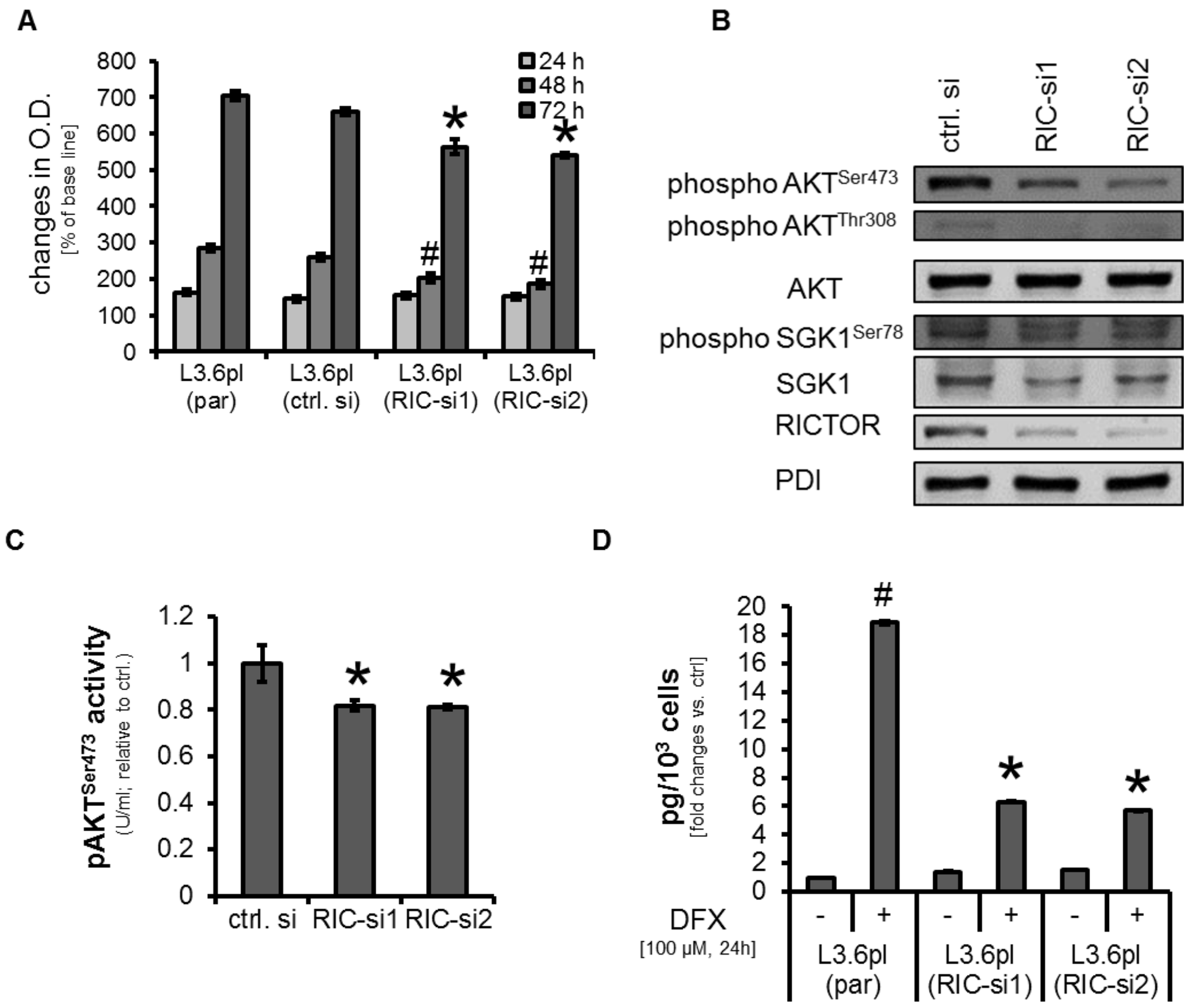

Supplementary Figure 2: Transient RICTOR knock-down in L3.6pl pancreatic cancer cells. (**A**) Transient transfection with RICTOR siRNA impairs tumor growth after 48 and 72 hours (#,*$p < 0.05$ vs. ctrl. si and par; bars = SEM). (**B**) RICTOR knock-down leads to inhibition of AKTSer473 phosphorylation. Furthermore, SGK1Ser78 phosphorylation is impaired. (**C**) Inhibition of AKTSer473 phosphorylation is confirmed by AKT activity assay (*$p < 0.05$ vs. ctrl. si). (**D**) DFX (100 µM, 24 h) induces VEGF-A secretion from L3.6pl cells ($^{\#}p < 0.05$). Targeting RICTOR significantly decreases DFX-induced VEGF-A secretion (*$p < 0.05$ vs. ctrl. si).

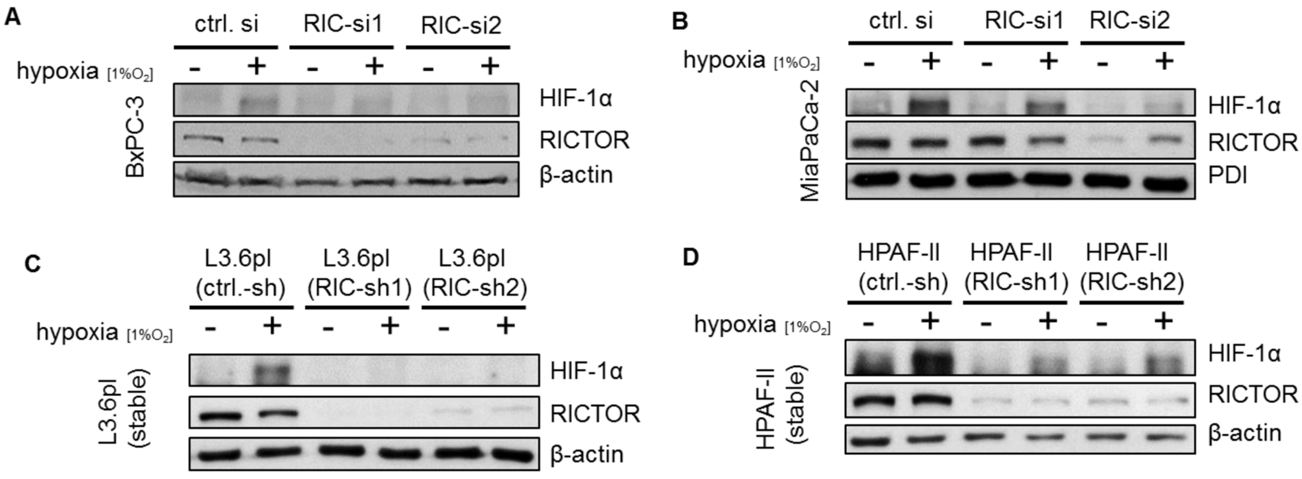

Supplementary Figure 3: Impact of RICTOR inhibition on HIF-1α expression upon "real" hypoxia. (**A–D**) Hypoxia (1%O2, 24 h) induces HIF-1α expression in all cell lines. RICTOR blockade with either transient (BxPC3 (A), MiaPaCa2 (B)) or stable (L3.6pl (C), HPAF-II (D)) knock-down efficiently reduces HIF-1α expression upon hypoxia conditions. In MiaPaCa-2, RICTOR knock-down was only found upon transfection with 1 siRNA (RIC-si2). Consequently, effects on HIF-1α expression after transfection with RIC-si1 were only marginal (B).

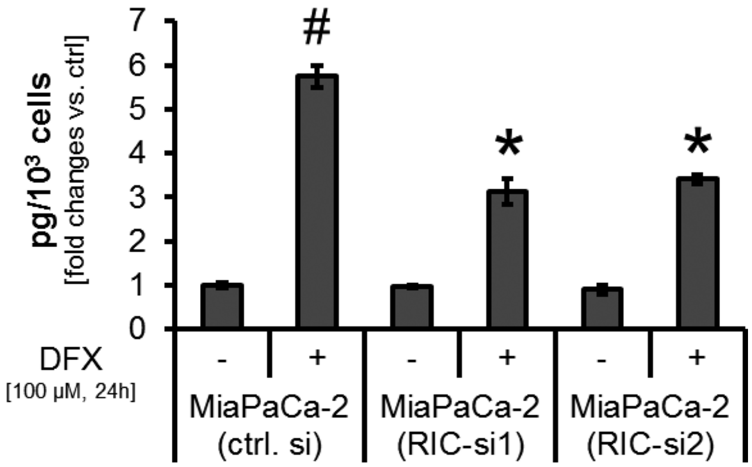

Supplementary Figure 4: Effect of transient RICTOR knock-down on VEGF-A secretion in MiaPaCa2 pancreatic cancer cells. DFX (100 µM, 24 h) induces VEGF-A secretion from MiaPaCa-2 cells ($^{\#}p < 0.05$). Targeting RICTOR significantly decreases DFX-induced VEGF-A secretion ($^{*}p < 0.05$ vs. ctrl. si).

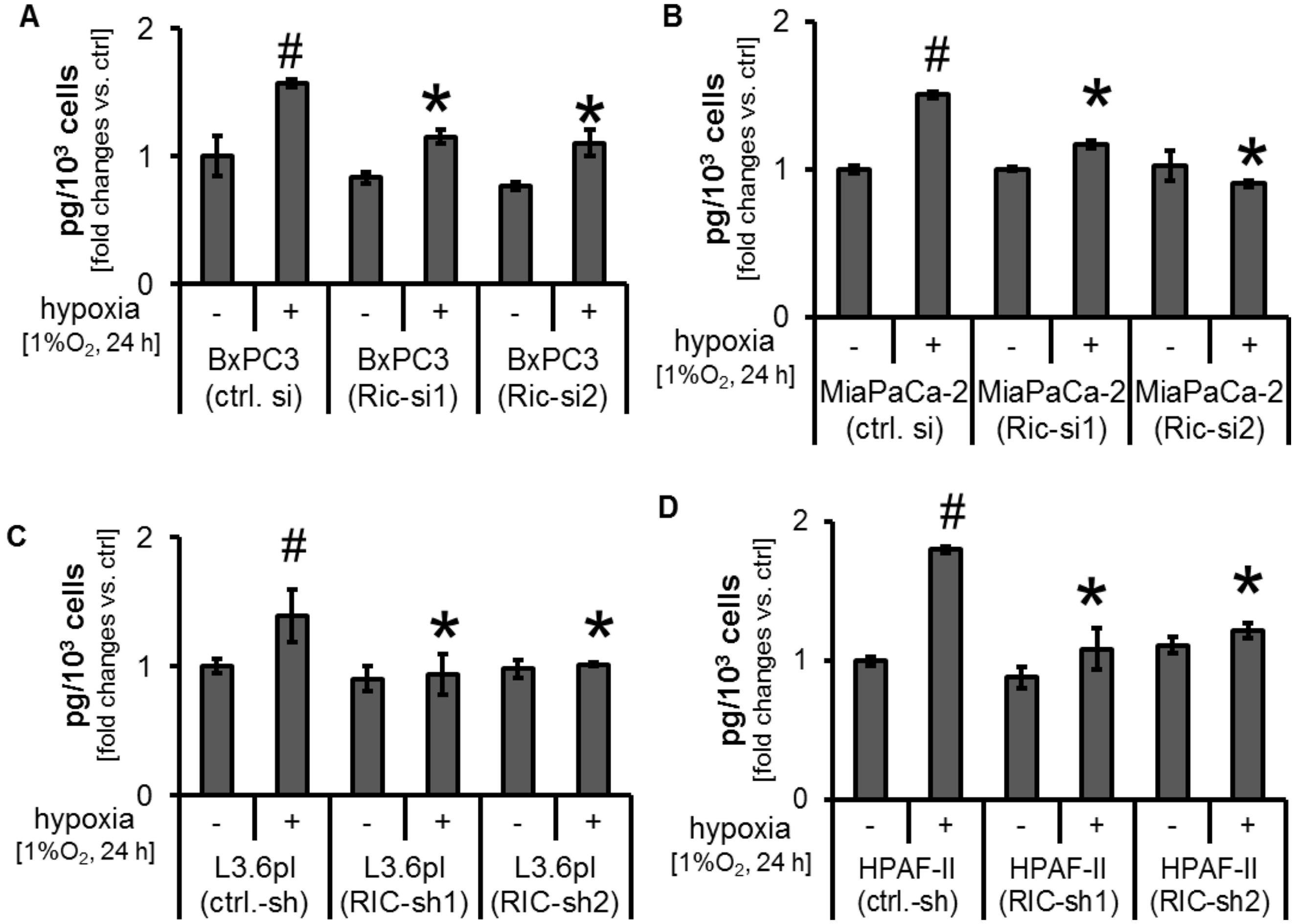

Supplementary Figure 5: VEGF-A secretion from human pancreatic cancer cell lines upon hypoxic conditions and RICTOR blockade. Hypoxia (1%O2, 24 h) induces VEGF-A secretion from BxPC3, MiaPaCa-2, L3.6pl and HPAF-II cells ($^{\#}p < 0.05$). Targeting RICTOR either by transient (**A, B**) or stable (**C, D**) knock-down significantly impairs secretion of VEGF-A ($^{*}p < 0.05$ vs. ctrl. si and ctrl.-sh).
